# Fish with slow life‐history cope better with chronic manganese exposure than fish with fast life‐history

**DOI:** 10.1002/ece3.70134

**Published:** 2024-08-08

**Authors:** Silva Uusi‐Heikkilä, Jouni K. Salonen, Juha S. Karjalainen, Ari Väisänen, Johanna Hippeläinen, Teemu Hämärvuo, Anna Kuparinen

**Affiliations:** ^1^ Department of Biological and Environmental Science University of Jyväskylä Jyvaskyla Finland; ^2^ Department of Chemistry University of Jyväskylä Jyvaskyla Finland

**Keywords:** feeding behavior, growth, life‐history type, manganese sulfate, standard metabolic rate, stress coping

## Abstract

Animals with different life‐history types vary in their stress‐coping styles, which can affect their fitness and survival in changing environments. We studied how chronic exposure to manganese sulfate (MnSO_4_), a common aquatic pollutant, affects life‐history traits, physiology, and behavior of zebrafish (*Danio rerio*) with two life‐history types: fast (previously selected for fast juvenile growth, early maturation, and small adult body size) and slow life histories (selected for slow juvenile growth, late maturation, and large adult body size). We found that MnSO_4_ had negative effects on growth and condition factors, but the magnitude of these effects depended on the life‐history type. Individuals with fast life histories were more susceptible to MnSO_4_ than fish with slow life histories as they had lower growth rate, condition factor and feeding probability in high MnSO_4_ concentrations. Our results demonstrate that MnSO_4_ can impair fish performance, and life‐history variation can modulate the stress‐coping ability of individuals.

## Abstract 2

Animals with different life‐history types vary in their stress‐coping styles, which can affect their fitness and survival in changing environments. We found that MnSO_4_ had negative effects on growth and condition factors, but the magnitude of these effects depended on the life‐history type. Individuals with fast life histories were more susceptible to MnSO_4_ than fish with slow life histories as they had lower growth rate, condition factor and feeding probability in high MnSO_4_ concentrations.

## 1 INTRODUCTION

Animals experience various abiotic and biotic stressors in their environment and often many of them simultaneously. These stressors challenge individuals and require them to adjust their behavior and/or physiology (e.g., Killen et al., 2013; Kolonin et al., 2022; Øverli et al., 2006). Individuals vary in their reactions to stress, hence there is also variation in stress‐associated physiological and behavioral traits (Kolonin et al., 2022; Koolhaas et al., 1999). The variation in stress‐coping can be a consequence of the amount of trait variation individuals harbor (plasticity) and the level of sensitivity they have (Engell Dahl et al., 2021; Radley et al., 2015). This has been demonstrated not only among species but also within species. An example of the former comes from Lake Tahoe where larvae of a native fish species were shown to be better able to cope with increased ultraviolet radiation by increasing their pigmentation than those of non‐native species (Gevertz et al., 2012). Individual variation towards confinement stress has been demonstrated in rainbow trout (*Oncorhynchus mykiss*; Pottinger et al., 1992). Sticklebacks (*Gasterosteus aculeatus*) originating from different populations showed different stress reactions towards predator cue and confinement (Bell et al., 2010).

Different life‐history strategies can also affect individuals' ability to respond to stress. Animals with ‘fast life histories’ (fast juvenile growth, early maturation, small adult body size, and reduced life span) are suggested to invest less in functions that are not directly related to growth and reproduction yet require a considerable amount of energy, such as detoxification (Congdon et al., 2001). Thus, individuals with fast life histories can have lower stress‐coping capabilities than ones with ‘slow life histories’ (slow juvenile growth, late maturation, large adult body size, and extended life span). This hypothesis has been tested only by a handful of studies. In an experiment where four damselfly species were exposed to pesticides, Debecker et al. (2016) demonstrated a stress response in the fastest‐living species, in terms of pesticide‐induced effect on the covariance between life history and boldness. Fast‐growing sticklebacks exposed to thermal stress had higher stress response in oxidative DNA damage (Kim et al., 2018) and birds with fast life histories suffered more from oxidative stress than birds with slow life histories (Vágási et al., 2019). Humans are changing environment at an increasing pace and exposing animals to different extrinsic stressors (Ceballos et al., 2015; Coleman & Williams, 2002; Häder et al., 2020). Therefore, it is important to understand individual variation in stress‐coping styles, what might be the mechanisms underlying these and what are the consequences of stress for populations consisting of individuals with, for example, different life‐history types. Indeed, stress coping is of fundamental importance to fitness and understanding individual differences in coping ability has become a paramount task in stress research (e.g., Bartolomucci et al., 2005; Cavigelli & McClintock, 2003; Korte et al., 2005).

Heavy metal and mineral pollution in aquatic environments are a global threat to fish populations (e.g., Kakade et al., 2023; Zamora‐Ledezma et al., 2021). Particularly manganese (Mn) and manganese sulfate (MnSO_4_) are contaminants elevated in aquatic ecosystems due to anthropogenic activities, such as mining and metal industry (Arola et al., 2017, 2019), tilling of acid sulfate soils (Nyman et al., 2023), and from wastewater and sewage systems (Howe et al., 2004). Short‐term exposures to Mn and MnSO_4_ have been shown to reduce growth in brown trout (*Salmo trutta*) at early life stages (Stubblefield et al., 1997) and increase larval and egg mortality in rainbow trout and whitefish (*Coregonus lavaretus*; Arola et al., 2017; Lewis, 1976). Although at high concentrations Mn is known to be harmful for aquatic organisms (Barnhoorn et al., 1999; Howe et al., 2004; Pinsino et al., 2010; Stubblefield et al., 1997) very little is known about the long‐term effects of moderately elevated (i.e., sublethal) concentrations of Mn to fish life histories, physiology, and behavior. Mn occurs naturally in surface waters at concentrations of 0.01–1.0 mg L^−1^ (Lydersen et al., 2002) and the long‐term freshwater environmental quality standards for Mn determined by species sensitivity distribution varies from 0.073 (0.033–0.466) mg L^−1^ in acid soft waters in Australia (Harford et al., 2015) to 0.062–0.123 mg L^−1^ in the UK (Peters et al., 2010).

In this study, we demonstrate long‐term effects of elevated concentration of MnSO_4_ to various phenotypic traits in fish with fast and slow life histories. To study the mechanisms of potential effects of MnSO_4_ on growth and condition factors, we additionally investigated its effects on physiology (metabolic rate) and behavior (activity and feeding). We used zebrafish (*Danio rerio*) populations which had been selected for body size (Uusi‐Heikkilä et al., 2015). This directional selection resulted in two different life‐history types: fast life‐history fish (selected for small body size; hereafter small‐selected fish) and slow life‐history fish (selected for large body size; hereafter large‐selected fish). To understand the effect of size selection on fish ability to cope with abiotic stress, we exposed both small‐ and large‐selected fish to manganese sulfate in a laboratory environment and monitored them from embryo to adulthood.

## 2 METHODS

### 2.1 Experimental design

The experimental fish originated from wild zebrafish (Uusi‐Heikkilä et al., 2010), which had been reared in the laboratory for 13 generations and from two different size‐selected populations with two replicates. Fish were selected for either large or small body size for five generations (Uusi‐Heikkilä et al., 2015). The size‐selective harvesting induced substantial phenotypic and genetic differences between the selection lines, which were consistent between the two selection‐line replicates (Uusi‐Heikkilä et al., 2015, 2017). Large‐selected fish had lower juvenile growth rates than small‐selected fish but reached higher maximum body size (L_∞_), had higher condition factors and reproductive success, and behaved differently (i.e., were more active, explorative, and bold; Uusi‐Heikkilä et al., 2015, 2017). Fish used in this experiment had recovered (i.e., no harvesting) eight generations from size selection. Although some phenotypic differences might have eroded during the recovery period, there were still large‐scale genetic differences between the selection lines (Uusi‐Heikkilä et al., 2017) and certain behavioral and cognitional differences between the selection lines have been shown to remain after up to 10 generations of recovery (Roy et al., 2023; Sbragaglia et al., 2019). The selection line refers to the life‐history type and we use these two terms interchangeably (small‐selected fish = fast life‐history type and large‐selected fish = slow life‐history type). Selection‐line replicate refers to the two replicated populations within a selection line.

We used 10 females and 20 males from each selection‐line replicate as parental fish. One female and two males were placed in a spawning tank and eggs were collected over 5 days. Embryos (and subsequently larvae and adult fish) were exposed to five different concentrations of MnSO_4_: 0.17, 0.5, 1.5, 3.5, and 7.5 mg L^−1^. Embryos of the control treatment were kept in tap water (approximately 0.02 Mn mg L^−1^). In preliminary experiments with concentrations of MnSO_4_ higher than 7.5 mg L^−1^, larval survival was extremely poor and therefore this was the highest concentration we utilized in this experiment. After hatching, larvae were moved to rearing tanks and fed daily with dry food (TetraMin) and Rotifers. Rearing temperature was kept at 26–27°C and the water exchange took place once a week.

At the age of 70 days post fertilization (dpf), fish were considered robust enough to survive from handling and measuring. For that, five individuals per selection‐line replicate were moved to rearing containers, which were placed in 30 L aquaria with eight containers in each (Figure S1). Because of uneven hatching and larval survival rate, the number of containers slightly varied per selection line per concentration. From now on, fish in the rearing containers are referred to as “rearing group”:

Despite weekly water exchange, Mn concentrations measured from the rearing water were low compared to the nominal concentrations (Table 1) likely because Mn accumulated in fish and absorbed on the surfaces of the aquaria and filters. The observed Mn concentrations (0.19 and 0.41 mg L^−1^) in our highest exposure treatments were, however above the recommended chronic no‐effect concentrations (Harford et al., 2015). Sulfate concentrations in our experiments remained well below the predicted no‐effect concentration of sulfate (39 mg L^−1^) in soft freshwaters (Karjalainen et al., 2023).

**TABLE 1:** Nominal concentrations of MnSO_4_, Mn and SO_4_ (mg L^−1^) and the average measured Mn concentrations (mg L^−1^) during the experiment.

Nominal concentrations			Measured concentrations
MnSO_4_ mg L^−1^	Mn mg L^−1^	SO_4_ mg L^−1^	Mn mg L^−1^
0.17	0.06	0.11	0.01
0.50	0.18	0.32	0.03
1.50	0.55	0.95	0.05
3.50	1.27	2.23	0.19
7.50	2.73	4.77	0.41

More details about the experimental design can be found in the Supplementary Material.

### 2.2 Growth experiment

To explore the differences in growth, which we used as a proxy for fitness (e.g., Perez & Munch, 2010), between the selection lines, we measured the average growth rate of fish reared in different MnSO_4_ concentrations. The standard length (SL) of each fish was measured once a week to the nearest 0.1 mm. For that, fish were anesthetized and photographed, and SL was measured from the photos with ImageJ image processing software (www.imagej.nih.gov). The measurements started at the age of 70 dpf, when fish were robust enough for anesthesia, and continued weekly until the age of 161 dpf. Because fish at age 70 dpf were assumed to be too fragile to survive individual marking, we did not have individual‐level body size data but used rearing group averages for SL. A final body size (SL) together with wet mass (WM) was measured at the age of 218 dpf. Then, we calculated the relative condition factor (*K*) for each fish.

### 2.3 Behavioral experiment

To explore the mechanisms behind potential differences in growth between the selection lines and among concentrations, we also studied differences in fish activity and feeding behavior. We subsampled eight fish per selection line per concentration at age 91 dpf and conducted a behavioral experiment in three concentrations: control, low (0.5 Mn mg L^−1^) and high (3.5 Mn mg L^−1^). The test arena was a 15 L aquarium divided into two sections with a plastic, transparent divider and with a grid with 3 × 3 cm squares attached to the walls and the bottom. Canon EOS 80D systems camera was set to record the fish movement in an aquarium on both sides, while another camera (GoPro Hero +) was set to record the aquarium from the third dimension (above). Individual fish were first acclimatized for 5 min in a plastic container after which the container was removed, and the fish could swim freely. We recorded fish movements for 5 min. After that, the fish was removed from the test arena and placed in a 4 L aquarium for an hour, after which the experiment was repeated to study whether the behavior was repeatable. Finally, we measured the SL of the fish.

Approximately one month later, we conducted the feeding experiment using the same fish (age 119 dpf) as in the activity experiment. Fish were fasting 24 h before the experiment to increase their motivation to feed. Feeding behavior was monitored in a separate aquarium (volume 15 L) and the behavior was recorded with a Canon EOS 80D camera which was set to record the fish movements on the side wall of the aquarium for 5 min. Each fish was first acclimatized for 5 min, after which a small amount of dry food was carefully dropped on the surface of the water. We then monitored feeding probability (i.e., whether the fish fed during the feeding trial or not) for 5 min. After the experiment, the fish was removed from the test aquarium and returned to its original rearing aquaria. The experiment was repeated after another 24 h fasting period. Finally, the SL of the fish was measured.

### 2.4 Metabolic rate measurements

Mass‐specific metabolic rates (mg O_2_ per g fish WM per h) of adult zebrafish (age 217 dpf) were estimated from the oxygen consumption of the fish measured in an intermittent‐flow respirometer connected to a fiber‐optic oxygen sensor (Loligo Systems 3‐channel OXY‐4 sensors). Fish were fasting 24 h before the measurement as feeding increases oxygen consumption (Ferreira et al., 2019). Oxygen consumption was measured at 25.3°C. We placed one rearing group (*N* = 5) at a time in the respirometer chamber (mean volume 313.5 mL). Oxygen consumption was measured for 3 min with a 3‐minute flush and a 2‐min wait period over a 20–21 h period resulting in altogether a total of 120–130 observations per group per measurement period. The chambers were kept for the first 2 hours in light and the following 18–19 h in the dark. There are different ways to define standard metabolic rate (SMR) and maximum metabolic rate (MMR; e.g., Hvas & Oppedal, 2019; Lucas et al., 2016; Svendsen et al., 2017). Here, SMR was based on the mean of the three lowest oxygen consumption values during the measurement period in the dark while MMR was assumed as the mean of the three highest oxygen consumption values in light. To exclude potential bacterial oxygen consumption, the oxygen consumption of empty chambers was measured before and after each measurement with fish in the chambers. If the difference between these two measurements of bacterial oxygen consumption was more than 1 mg O_2_ per min, a linear interpolation was applied over the data. After the respirometer measurements, we measured the SL and WM of each fish.

### 2.5 Manganese concentration analyses

We explored Mn accumulation to better understand the effects of Mn exposure on fish. Single organs (e.g., liver, brain) were too small to provide sufficient dry weight for Mn detection. Instead, we focused on the head (e.g., brain, gills) and the rest of the body (e.g., liver, muscle tissue), i.e., we used two samples per fish in the analyses. Samples were freeze dried for 48 h (Christ Alpha 2–4), smashed and digested in aqua regia (HNO3:HCl 1:3). Then, the digested tissue samples were filtered and filled to a final volume of 10 mL with ultrapure water, after which the samples were analyzed similarly than the water samples with Perkin‐Elmer Avio 500 ICP OES.

### 2.6 Statistical analyses

To reveal the potential effects of MnSO_4_ concentration and selection line on fish growth, we extracted the average relative growth rate of the rearing groups with

$${\left({\mathrm{SL}}_{2}/{\mathrm{SL}}_{1}\right)}^{\left(1/t\right)}-1$$

where SL_2_ is the average SL at the age 218 dpf (the last measurement), SL_1_ is the average SL at the age 70 dpf (the first measurement) and *t* is the time, the experiment lasted (148 days). We then used a generalized additive model (GAM) with Gaussian error distribution and maximum likelihood estimation with the relative growth rate and condition factor as response variables and concentration, selection line, and their interaction as predictive variables (1). Selection‐line replicate was not set as a random variable because in some concentrations, we only had one rearing group per selection‐line replicate. As fish SL varied among concentrations and selection lines at the time we started the experiment, we additionally added the average body size at the time the growth experiment was initiated (SL at the age 70 dpf) as a predictive variable in the model (1). SL at the age 70 dpf was not explained by the selection line (*F* = 0.226, *p* = .637) or by the MnSO_4_ concentration (*F* = 0.014, *p* = .905). Growth rate was estimated as an average relative growth rate across all individuals within a rearing group, hence the rearing group could not be added as a random variable in the model.

(1) $$\begin{array}{c} \mathrm{GAM}\;(\text{Growth rate}\sim \text{Selection line}\times \text{Concentration}\\ +\mathrm{SL}\;\mathrm{at}\;\mathrm{age}\;70\;\mathrm{dpf},\text{family}=\text{Gaussian}) \end{array}$$

Condition factor, on the other hand, was calculated for each individual fish at the end of the experiment (at age 218 dpf), not as rearing group averages as when estimated growth rates. Therefore, the effect of rearing group could be added as a random factor in the model (edf = 2.611, *p* = .108). Selection‐line replicate (both selection lines had two replicates) could not be added as a random variable as in some concentrations we only had five fish, i.e., one rearing group, per selection‐line replicate. The relative condition factor (Froese, 2006; Le Cren, 1951) was calculated for each fish as

$$\mathrm{WM}/\left(a\times {\mathrm{SL}}^{b}\right)$$

where WM is the wet mass (g) at age 218 dpf, SL is the standard length (mm) at age 218 dpf, *a* is the intercept and *b* is the slope of a linear regression of ln(WM) on ln(SL) (*t* = 5.406, *p* < .01). The standard length–weight regression parameters of our zebrafish population were estimated as *a* = 0.0555 and *b* = 2.619 using fish in the control treatment (i.e., 0 Mn mg L^−1^).

(2) $$\begin{array}{c} \mathrm{GAM}\;\text{Condition factor}\sim \text{Selection line}\times \text{Concentration}\\ +\mathrm{SL}\;\mathrm{at}\;\mathrm{age}\;70\;\mathrm{dpf}+\left(1|\text{Rearing group}\right),\text{family}=\text{Gaussian} \end{array}$$

Behavioral traits (activity and feeding probability) were tested twice to demonstrate consistency in fish behavior over time. This was studied with a correlation test between the two measurements per individual fish. We used a (generalized) linear mixed‐effect model (G/LMER) to study the effects of MnSO_4_ concentration and selection line on fish behavior. Activity (distance moved in cm) and feeding behavior (feeding probability) were set as response variables and selection line, MnSO_4_ concentration, their interaction, and fish SL at the time of measuring the behavior as predictive variables (3,4). Individual fish (as each fish was measured twice) and rearing group were set as random variables.

(3) $$\begin{array}{c} \text{LMER}\;(\text{Activity}\sim \text{Selection line}\times \text{Concentration}\\ +\mathrm{SL}+\left(1|\text{Fish}\;\mathrm{ID}\right)+\left(1|\text{Rearing group}\right)) \end{array}$$

(4) $$\begin{array}{c} \text{GLMER}\;(\text{Feeding probability}\sim \text{Selection line}\times \text{Concentration}\\ +\mathrm{SL}+\left(1|\text{Fish}\;\mathrm{ID}\right)+\left(1|\text{Rearing group}\right),\text{family}=\text{Binomial}) \end{array}$$

A generalized additive model was used to study the effects of MnSO_4_ concentration and selection line (predictive variables) on average metabolic rates (SMR and MMR) and aerobic scope (response variables). As metabolic rates were estimated for groups of fish originating from one rearing group, it could not be added as a random variable in the model (5). The number of fish in one respirometer chamber during the measurement varied slightly (i.e., we did not always have exactly five fish in a chamber at a time) and therefore the effect of number of fish in a chamber during the measurement could be tested as adding it as a random variable (edf = 0.5927, *p* = .122).

(5) $$\begin{array}{c} \mathrm{GAM}\;(\mathrm{SMR}/\mathrm{MMR}/\mathrm{AS}\sim \text{Selection line}\\ \times \text{Concentration}+\left(1|\text{Number of fish in chamber}\right)) \end{array}$$

Finally, we used a linear model (LM) to study the effects of MnSO_4_ concentration and selection line (predictive variables) on Mn‐concentration (mg Mn/g fish WM) accumulated in the fish head and in the body (6).

(6) $$\mathrm{LM}\;\left(\mathrm{Mn}-\text{concentration}\sim \text{Selection line}\times \text{Concentration}\right)$$

To estimate differences in growth rate, condition factor, standard metabolic rate, behavior and Mn accumulation in fish head and body in different Mn concentrations and between the selection lines, we first fitted the full model and then used the stepwise model reduction. Results were considered statistically significant at *p* < .05. Data was analyzed using R version 4.1.2 (R Core Team, 2022) and packages nlme, lme4, mgcv and pscl.

## 3 RESULTS

### 3.1 The effect of MnSO_4_ concentration and selection line on growth and condition

**FIGURE 1:**
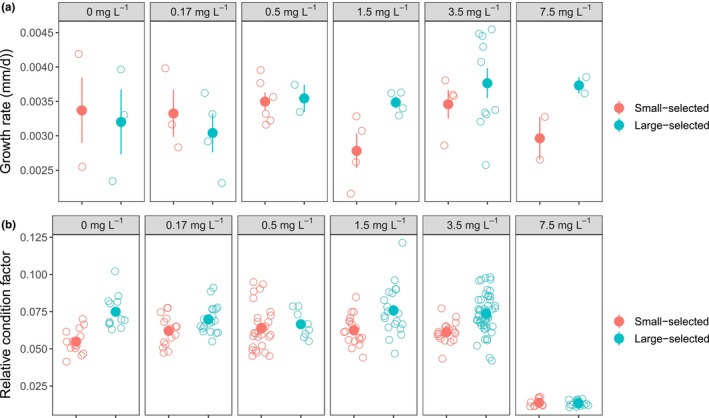
(a) The average relative growth rate of fish from the small‐selected (red symbols) and large‐selected (turquoise symbols) fish at different MnSO_4_ concentrations (gray bars on top; Mn mg L^−1^). Data are shown as the average relative growth rate (open circles) per rearing cage and the mean (filled circles) with standard errors. (b) Relative condition factor at age 218 days post fertilization of the small‐selected (red symbols) and large‐selected (turquoise symbols) fish at different MnSO_4_ concentrations. Data are shown as individual observation (open circles) per fish and the mean (filled circles) with standard errors.

Large‐selected fish had higher average growth rates than small‐selected fish (*t* = 2.546, *p* = .011; Figure 1a, Table S1) and fish also had lower growth rates at high concentrations compared to low concentrations (*t* = 2.062, *p* = .039; Table S1), particularly the small‐selected fish (Figure 1a). The average body size at the beginning of the experiment (SL at age 70 dpf) also significantly affected the growth rate (*t* = −16.72, *p* < .001; Table S1). Generally, the smaller the fish when the experiment started, the higher the relative growth rate.

The relative condition factor at age 218 dpf was significantly affected by the interaction of the selection line and concentration (t = −1.983, *p* = .023; Table S1). Large‐selected fish had higher condition factors than small‐selected fish in all concentrations except in 0.5 and 1.5 Mn mg L^−1^ (Figure 1b). Among both selection lines, the condition factor was clearly lowest in the highest MnSO_4_ concentration compared to the other concentrations (Figure 1b). Similarly, as in the growth experiment, the size of the fish when the experiment started (at age 70 dpf) had an effect on the adult condition factor: the smaller the fish when the experiment started the higher the condition factor at the end of the experiment (t = −4.686, *p* < .001; Table S1).

### 3.2 The effect of MnSO_4_ concentration and selection line on behavior

**FIGURE 2:**
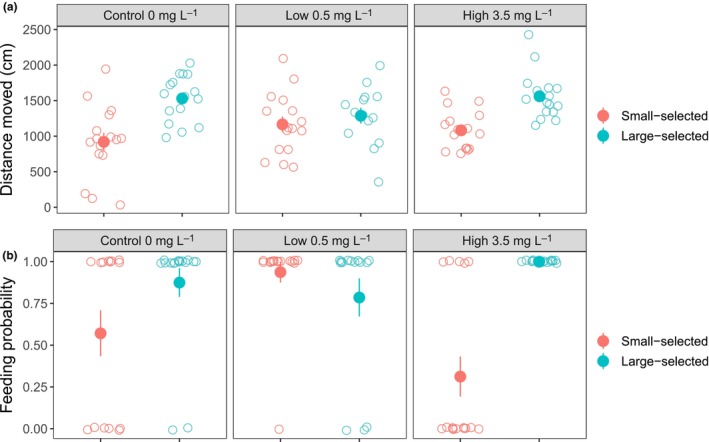
The effect of MnSO_4_ concentration and selection line on (a) general activity measured as a distance moved (cm) and (b) feeding activity measured as feeding probability during the five‐minute behavioral trial. Small‐selective fish indicated by red symbols and large‐selected fish by turquoise symbols. Data are shown as individual observation (open circles) per fish and the mean (filled circles) with standard errors.

The activity was a repeatable behavioral trait (Pearson's correlation = 0.437; *p* = .002). There was no significant interaction between the selection line and concentration neither concentration affected fish activity (Figure 2a, Table S2). Large‐selected fish had overall higher activity levels than small‐selected fish (*t* = 2.633, *p* < .01; Figure 2a, Table S2) and larger fish had higher activity levels irrespective of the selection line (*t* = 2.988, *p* = .006; Table S2).

Feeding probability was also a repeatable behavioral trait (Kendall’s tau = 0.479; *p* = .001). Among small‐ and large‐selected fish, 22% and 9% of the fish, respectively, did not feed during the feeding trial. The interaction between the selection line and concentration explained differences in feeding probability (*z* = 1.580, *p* = .002; Table S2). While among large‐selected fish the feeding probability increased with MnSO_4_ concentration, among small‐selected fish the feeding probability slightly increased first but then decreased distinctively in the highest concentration (Figure 2b). Fish body size did not affect feeding probability (Table S2).

### 3.3 The effect of MnSO_4_ concentration and selection line on physiology

The standard metabolic rate (Figure S2A), maximum metabolic rate (Figure S2B) and aerobic scope (Figure S2C) were not affected either by the selection line or by MnSO_4_ concentration (Table S3).

### 3.4 The effect of MnSO_4_ concentration and selection line on accumulated Mn concentration in tissues

**FIGURE 3:**
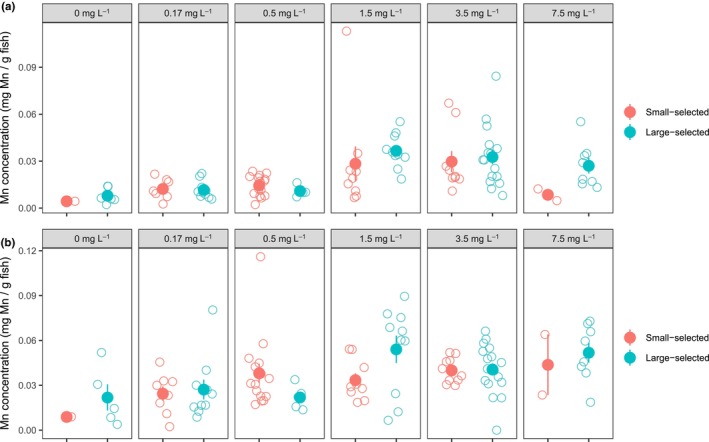
The effect of MnSO_4_ concentration in the rearing environment and selection line on the accumulated amount of manganese in the (a) body and (b) head of the fish. Small‐selective fish indicated by red symbols and large‐selected fish by turquoise symbols. Data are shown as individual observation (open circles) per fish and the mean (filled circles) with standard errors.

Manganese tended to accumulate more in the head than in the body of the fish (Figure 3a,b). The selection line had no significant effect on Mn concentration either in the head or in the body (Table S4), however the higher the Mn concentration in the water the higher it was in the body (*t* = 12.47, *p* = .003; Figure 3a) and in the head (*t* = 17.67, *p* = .001; Figure 3b).

## 4 DISCUSSION

Life‐history type (i.e., selection line) affected fish performance under stressful environmental conditions. Large‐selected fish, which had been selected for large body size, slow juvenile growth rate and old age at maturity (“slow life‐history type”) seemed to tolerate better high MnSO_4_ concentrations than small‐selected fish with small body size, fast juvenile growth rate and young age at maturity (“fast life‐history type”; Uusi‐Heikkilä et al., 2015). Large‐selected fish had higher growth rate and condition factor in elevated MnSO_4_ concentrations than small‐selected fish despite no significant differences in standard metabolic rate. They were also more active and had higher feeding probability. These results suggest, that potentially owing to different energy allocation strategies, personalities or stress coping styles, individuals with slow life histories may be better able to cope with chronic environmental stress. Therefore, we should react with caution to statements that a certain stress response is common across all individuals within a species (Balasch & Tort, 2019). Instead, it may depend on individual characteristics, such as life‐history types or personalities (Caizergues et al., 2022; Prentice et al., 2022; but see Santicchia et al., 2020).

Although manganese accumulated in a similar manner to fish with both life‐history types (Figure 3), the growth response differed between them. Fish with a selection history for slow life performed better than fish with a selection history for fast life in terms of growth (Figure 1). The difference was absent in the control treatment, which indicates recovery of this trait from the past selection but became visible when individuals experienced more stressful environments (i.e., elevated MnSO_4_ concentrations). Previous study has shown that large‐selected fish exhibit more variation in growth and respond to starvation stress differently than small‐selected fish (Uusi‐Heikkilä et al., 2016). These observations imply that large‐selected fish are more plastic and better able to cope with environmental stressors than small‐selected fish. Indeed, it has been suggested by other studies that stress‐coping abilities are affected by the degree of individual phenotypic plasticity (Balasch & Tort, 2019).

The mechanisms behind the higher growth rate of large‐selected fish may be related to differences in, for example, stress physiology and/or energy metabolism. We did not detect any significant trends in the standard metabolic rate, which neither differed between the life‐history types nor was linearly affected by the MnSO_4_ concentration. This finding could be related to the small sample size, high variation, as fish metabolic rates were measured in groups, and/or to the age of measured fish (i.e., they had already exceeded the period of fast growth). Furthermore, as the MnSO_4_ concentrations could not be controlled in the respirometer chambers, the measurements were done in pure water. In addition to the metabolic rate, there might be other physiological traits (e.g., feed conversion ratio or metabolic efficiency) that could explain the higher growth rate of large‐selected fish in high MnSO_4_ concentrations.

The effective Mn concentrations in our long‐term experiment (measured mean concentrations in water 0.19 and 0.41 mg L^−1^, nominal concentrations 1.27 and 2.73 mg L^−1^) were higher than the predicted no‐effect concentrations for aquatic organisms recommended (Harford et al., 2015; Peters et al., 2010). Acute effective or lethal concentrations of Mn are clearly higher, for example, the effective concentration (EC10) of zebrafish embryos is 4.63 mg L^−1^ (Peters et al., 2010). Mn concentration of more than 4.5 mg L^−1^ (10 times higher than our highest measured concentration) has been shown to decrease growth in brown trout early stages (*Salmo trutta*) and concentrations higher than 15.5 mg L^−1^ can be lethal (Stubblefield et al., 1997). Interestingly, even very high Mn concentration (100 mg L^−1^) did not affect brown trout hatching success (Stubblefield et al., 1997). Arola et al. (2017) demonstrated slightly lower concentrations (approx. 15–30 mg L^−1^) as LC50 value for whitefish (*Coregonus lavaretus*) offspring, whereas Lewis (1976) noticed only 1 mg L^−1^ MnSO_4_ to increase embryonic mortality in rainbow trout. Several studies have demonstrated different sublethal physiological effects of Mn in fish, for example, altered hematological parameters (Aliko et al., 2018), such as decreased number of red blood cells and hemoglobin value (Sharma & Langer, 2014) even without mortality itself (Wepener et al., 1992). Furthermore, the toxicity level of Mn is suggested to be associated with oxidative stress (Vieira et al., 2012; but see Baden et al., 1995). Increase in water temperature also increases the uptake of Mn in fish (Howe et al., 2004), also raising the potential effect of climate‐induced changes in water temperature in this context.

Large‐selected fish were not only able to grow faster but they were also able to potentially allocate more energy into fat production particularly in high concentrations indicated by higher condition factors (Figure 1b). This process was, however, severely disrupted in the highest concentration (7.5 mg L^−1^) where the condition factor of both large‐ and small‐selected fish was five times lower than in other concentrations and could reflect altered metabolic homeostasis. In the highest concentration, there were more individuals with very low than high condition factors among both life‐history types. One explanation underlying this bimodal distribution could be related to social structure in zebrafish shoals. In stressful conditions, the competition for food might become more intense as energy demand increases and creates more pronounced social hierarchies where few individuals dominate the resources. Among large‐selected fish, there seemed to be more of these potentially dominating individuals than among small‐selected fish. Similar decreases in condition factors in fish exposed to high heavy metal concentrations have been reported earlier because of reduced feed intake or metabolic activity (Baudou et al., 2017; Eastwood & Couture, 2002; but see Dethloff et al., 2001; Farag et al., 1995). While the effect of environmental toxins and heavy metals on fish condition has been well studied and demonstrated, less attention has been paid to the interaction between heavy metal concentrations and individual differences in life histories. Therefore, it is important to consider the heterogeneity of stress response among individuals in an environment where several human‐induced selection pressures operate simultaneously and potentially antagonistically (e.g., size‐selective harvesting and heavy metal exposure).

Energy demand typically increases under stress and growth may be compromised if the animal cannot balance their energetic requirements (Rueda‐Jasso, 2004). Indeed, toxic agents may lead to an imbalance between energy supply and demand by negatively affecting feeding behavior either directly or indirectly (e.g., damaging sensory, and/or nervous system; Hoskins & Volkoff, 2012). Studies have reported reduced intake of food in cadmium‐exposed fish together with altered swimming activity (Baudou et al., 2017; Ferrari et al., 2011; Sloman et al., 2003). In fish, appetite is commonly associated with increased swimming activity as they search for food. The lower feeding probability (Figure 2b) likely underlies, at least partly, the lower growth rate and condition factor of small‐selected fish compared to large‐selected fish in our study. Small‐selected fish were also less active (Figure 2a), and this could indicate that they were not searching food as effectively as large‐selected fish or alternatively they were saving energy. This, in turn, could indicate that they had lower appetite. However, differences in feeding behavior and activity between the two life‐history types were already present in the control treatment. Large‐selected fish have also been previously shown to be more active and explorative than small‐selected fish (Uusi‐Heikkilä et al., 2015), thus it seems that these behavioral differences are correlating with other morphological and life‐history differences characterizing these different life‐history types.

Behavioral responses to stress have been described as reactive (often characterized by freezing behavior) or proactive (e.g., highly active fight or flight behavior). In high MnSO_4_ concentrations, small‐selected fish appeared to adopt a reactive behavior (less active, low feeding probability) whereas large‐selected fish behaved evidently proactively (more active, high feeding probability). These behavioral types are considered as adaptations for life in unstable and stable environments, respectively; thus, reactive individuals are characterized by higher levels of physiological stress responses than proactive individuals (Cockrem, 2007). In vertebrates, the physiological stress response involves activation of the hypothalamic–pituitary–adrenal (HPA) axis, where exposure to stress stimulates the secretion of glucocorticoids (e.g., Cockrem, 2007). In turn, glucocorticoid secretion elicits a cascade of physiological and behavioral processes that are essential to cope with stressful events (Landys et al., 2006; Wingfield & Ramenofsky, 1999). The stress‐coping style hypothesis makes the specific predictions that proactive individuals should have lower baseline concentrations of glucocorticoids and a less reactive HPA axis. Although we did not measure cortisol concentrations of the fish in the present study, we have shown earlier that cortisol concentration in zebrafish correlates negatively with body size and feed intake (Merino et al., unpublished; Uusi‐Heikkilä et al., 2018).

Finally, small‐selected fish have been shown to differ genetically (both at sequence and gene expression levels) from large‐selected fish (Uusi‐Heikkilä et al., 2015, 2017). Although gene expression profiles of the experimental fish were not investigated in the present study, it is possible that there were differences in certain stress‐related regulatory mechanisms between the life‐history types leading to differences in the ability to compensate the effects of the stressor. This type of a response is often associated with chronic stress since heavy acute stressors may result in death, and mild ones in recovery.

In the present study, we demonstrated that life‐history type may affect stress‐coping ability in fish. Considering the complex response to stress becomes important when human activities are imposing different selection pressures on wild animal populations. For example, fisheries is often size‐selective and select for small body size and fast life‐history types (e.g., Jørgensen & Fiksen, 2010; Olsen et al., 2004; Uusi‐Heikkilä et al., 2015). Harvested populations may experience other human‐induced selection pressures in their environment operating, for example, on stress coping abilities. Therefore, if a population mostly consists of individuals with fast life‐histories and potentially low stress‐coping abilities, the two selection pressures operating antagonistically may exacerbate population decline. It is also good to keep in mind that human‐induced selection pressures are often directional and may reduce plasticity in a population, which has been suggested to help coping with maladaptive stressors (Balasch & Tort, 2019). While complicating the predictions of how organisms may respond to stress, these are important factors to consider when anticipating the effects of multiple, simultaneous human‐induced stressors on heterogeneous populations.

## AUTHOR CONTRIBUTIONS

**Silva Uusi‐Heikkilä:** Conceptualization (equal); data curation (equal); formal analysis (lead); investigation (equal); methodology (equal); supervision (equal); visualization (lead); writing – original draft (lead); writing – review and editing (equal). **Jouni K. Salonen:** Conceptualization (equal); data curation (equal); investigation (equal); methodology (equal); supervision (equal); writing – original draft (equal); writing – review and editing (equal). **Juha S. Karjalainen:** Data curation (supporting); investigation (supporting); resources (supporting); writing – review and editing (equal). **Ari Väisänen:** Data curation (supporting); investigation (supporting); methodology (supporting); resources (supporting); writing – review and editing (equal). **Johanna Hippeläinen:** Conceptualization (supporting); formal analysis (supporting); investigation (supporting); methodology (supporting); writing – review and editing (equal). **Teemu Hämärvuo:** Data curation (supporting); formal analysis (supporting); investigation (supporting); writing – review and editing (equal). **Anna Kuparinen:** Conceptualization (equal); formal analysis (supporting); funding acquisition (lead); project administration (lead); resources (lead); writing – review and editing (equal).

## FUNDING INFORMATION

This study was funded by the Academy of Finland (project grants 325107 to SUH and 317495 to AK), Emil Aaltonen Foundation (through a project grant to AK). This project has also received funding from the European Research Council (ERC) under the European Union's Horizon 2020 Research and Innovation Programme (grant agreement No 770884).

## CONFLICT OF INTEREST STATEMENT

The authors declare no competing interests.

## Supporting information


Data S1

Figure S1

Figure S2

## Data Availability

Data and codes are available in the Jyväskylä University Digital Repository (jyx.jyu.fi) upon publication.
