# Supplementary material for: Fish with slow life‐history cope better with chronic manganese exposure than fish with fast life‐history
Source: Ecol Evol. 2024 Aug 8;14(8):e70134. doi: 10.1002/ece3.70134 (PMC11307103; doi:10.1002/ece3.70134)
Supplement: Supplementary file 1 — Data S1 [file ECE3-14-e70134-s002.docx]

**Fish with slow life-history cope better with chronic manganese exposure than fish with fast life-history**

**SUPPLEMENTARY MATERIAL**

**METHODS**

**Experimental design**

After fish were exposed to MnSO_4_ at age 70 days post fertilization (dpf), they were fed twice a day with dry food and 20 L water exchange took place weekly. Water temperature (mean ± standard deviation: 26.5 ± 0.53) was monitored daily, pH was measured twice a week, and NO_2_-concentration (< 0.01 mg L^-1^) once a week. Mn toxicity is known to depend on water hardness and acidity: lower hardness (i.e., CaCO_3_ concentration) and pH lead to higher toxicity for aquatic organisms (Harford et al., 2015). The average pH was 7.58 ± 0.03 and water hardness was 0.21 mmol L^-1^ (CaCo3 hardness 21 mg L^-1^).

Mn concentrations in the rearing water were measured daily during the first two weeks, and twice a week after that. For the analyses, 10 ml water samples were taken and Perkin-Elmer Avio 500 inductively coupled plasma-optical emission spectrometry (ICP OES) was used. All the concentration measurements were carried out using a four-point calibration and multi element calibration standards were used for all elements. The measurements were performed by taking two most sensitive emission lines to attain the sensitivity required.

**SUPPLEMENTARY TABLES**

**Table S1.** The effect of selection line, MnSO_4_ concentration, and initial standard length (SL) on growth rate (mm/d) and relative condition factor. Significant *P*-values in bold. The reference selection line is small-selected.

| **Trait** | **Variable** | **Deviance** | ***P*-value** | **Variable estimate (s.e.)** |
| --- | --- | --- | --- | --- |
| **Growth** | Selection line × concentration | -2.2157e-09 | 0.8234 | -0.0000067 (0.000030) |
|  | Selection line | -2.8211e-07 | **0.0109** | 0.0001569 (0.0000616) |
|  | Concentration | -1.8501e-07 | **0.0392** | 0.0000293 (0.0000142) |
|  | SL at age 70 dpf | -1.1063e-05 | **< 0.001** | -0.0003675 (0.0000220) |
| **Condition factor** | Selection line × concentration | -0.12086 | 0.0228 | -0.025721 (0.013148) |
|  | Selection line | -0.90269 | **< 0.001** | 0.206889 (0.039714) |
|  | Concentration | -8.4458 | **< 0.001** | -0.072755 (0.009856) |
|  | SL at age 70 dpf | -0.687 | **< 0.001** | -0.047636 (0.010166) |

**Table S2.** The effect of selection line, MnSO_4_ concentration and standard length (SL) on activity (distance moved; cm) and feeding probability. Significant *P*-values in bold. The reference selection line is small-selected.

| **Trait** | **Variable** | **χ^2^-value** | ***P*-value** | **Variable estimate (s.e.)** |
| --- | --- | --- | --- | --- |
| **Activity** | Selection line × concentration | 0.0159 | 0.8997 | 5.686 (55.437) |
|  | Selection line | 12.086 | **< 0.001** | 309.028 (117.385) |
|  | Concentration | 0.6799 | 0.4096 | 18.383 (39.021) |
|  | Adult SL | 7.5258 | **0.005** | 85.709 (28.684) |
| **Feeding probability** | Selection line × concentration | 15.143 | **0.002** | 1.7170 (1.0869) |
|  | Concentration | 1.0136 | 0.314 | -0.7036 (0.3951) |
|  | Selection line | 4.4022 | **0.036** | 0.2629 (1.1328) |
|  | Adult SL | 3.3695 | 0.066 | 0.5146 (0.3961) |

**Table S3.** The effect of selection line and MnSO_4_ concentration on standard metabolic rate (SMR), maximum metabolic rate (MMR) and aerobic scope (mg O_2_/g/h). The reference selection line is small-selected.

| **Trait** | **Variable** | **Deviance** | ***P*-value** | **Variable estimate (s.e.)** |
| --- | --- | --- | --- | --- |
| **SMR** | Selection line × concentration | 8.0388e-05 | 0.1102 | -0.0049 (0.0093) |
|  | Selection line | -0.0019907 | 0.5961 | 0.0008 (0.0277) |
|  | Concentration | -0.0029833 | 0.5161 | 0.0001 (0.0069) |
| **MMR** | Selection line × concentration | -0.084602 | 0.268 | 0.0289 (0.0241) |
|  | Selection line | -0.019458 | 0.425 | -0.0867 (0.0722) |
|  | Concentration | -0.031875 | 0.532 | -0.0257 (0.0179) |
| **Aerobic scope** | Selection line × concentration | -0.107210 | 0.156 | 0.0350 (0.0247) |
|  | Selection line | -0.011644 | 0.641 | -0.0911 (0.0739) |
|  | Concentration | -0.018987 | 0.553 | -0.0267 (0.0183) |

**Table S4.** The effect of selection line and MnSO_4_ concentration on Mn-concentration (mg Mn / g fish) in the head and body of exposed fish. Significant *P*-values in bold. The reference selection line is small-selected.

| **Trait** | **Variable** | **Sum of squares** | ***P*-value** | **Variable estimate (s.e.)** |
| --- | --- | --- | --- | --- |
| **Concentration (head)** | Selection line × concentration | -0.0000962 | 0.627 | 0.0010 (0.0020) |
|  | Selection line | -0.0000502 | 0.724 | -0.0004 (0.0056) |
|  | Concentration | -0.0042740 | **0.001** | 0.0022 (0.0017) |
| **Concentration (body)** | Selection line × concentration | -0.0000508 | 0.680 | 0.0007 (0.0017) |
|  | Selection line | -0.0001839 | 0.430 | 0.0015 (0.0047) |
|  | Concentration | -0.0026295 | **0.003** | 0.0017 (0.0015) |

**SUPPLEMENTARY FIGURE LEGEND**

**Figure S1.** A schematic description of experimental design. One aquarium (illustrated by squares) per MnSO_4_ concentration with eight rearing containers each (illustrated by circles). Each rearing container was placed with five fish (N=5). SS1 = small-selected line (replicate 1), and SS2 = small-selected line (replicate 2), LS1 = large-selected line (replicate 1), and LS = large-selected line (replicate 2). D = dummy fish (random fish to maintain same density of fish, i.e., 40 individuals, per aquarium).

**Figure S2.** The effect of MnSO_4_ concentration and selection line on (A) standard metabolic rate, (B) maximum metabolic rate, and (C) aerobic scope. Small-selective fish indicated by red symbols and large-selected fish by turquoise symbols. Data are shown as average relative growth rate (open circles) per rearing cage and the mean (filled circles) with standard errors.
