# Supplementary figures and images for: Fish with slow life‐history cope better with chronic manganese exposure than fish with fast life‐history
Source: Ecol Evol. 2024 Aug 8;14(8):e70134. doi: 10.1002/ece3.70134 (PMC11307103; doi:10.1002/ece3.70134)

**0 mg L<sup>-1</sup>**

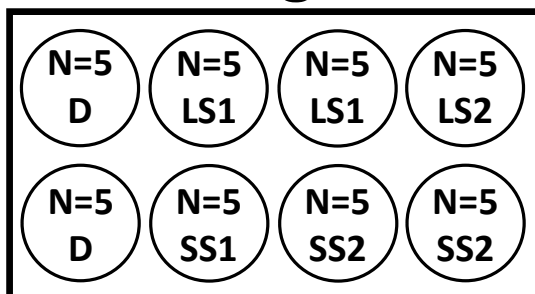

**0.17 mg L<sup>-1</sup>**

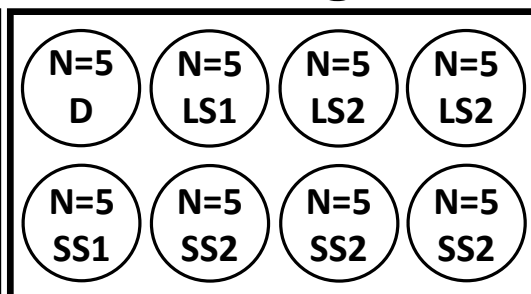

**0.5 mg L<sup>-1</sup>**

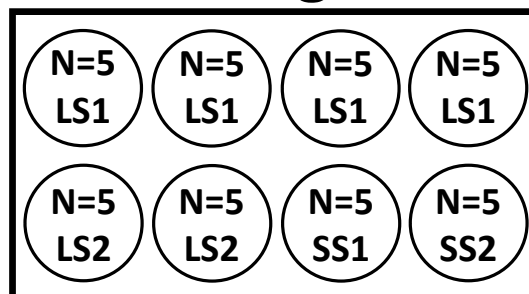

**1.5 mg L<sup>-1</sup>**

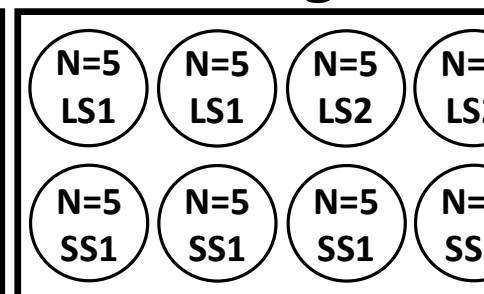

**3.5 mg L<sup>-1</sup>**

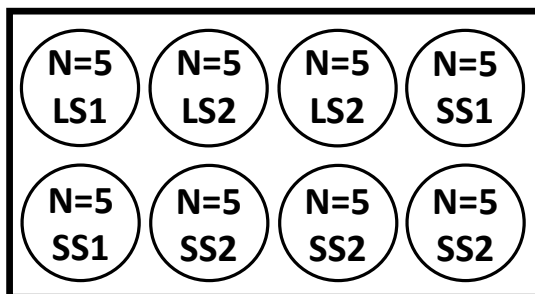

**3.5 mg L<sup>-1</sup>**

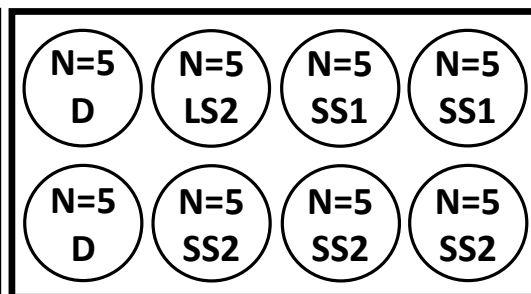

**7.5 mg L<sup>-1</sup>**

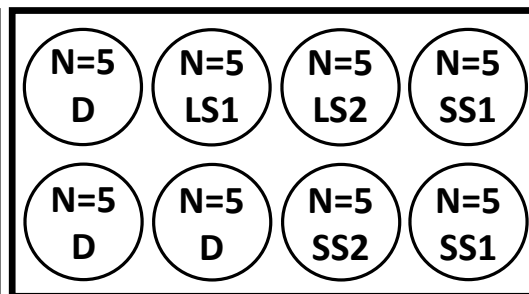

Supplement: Supplementary file 2 — Figure S1 [file ECE3-14-e70134-s003.pdf]

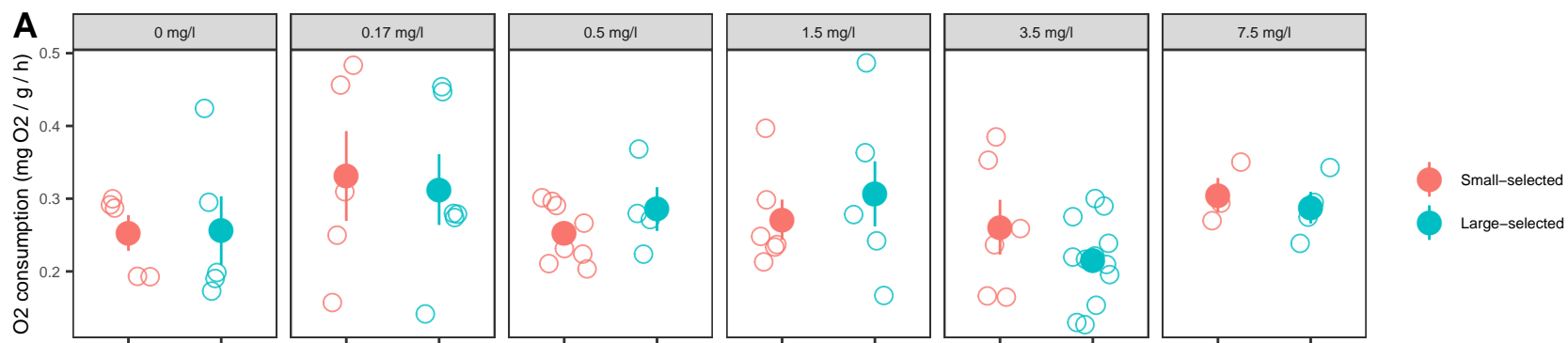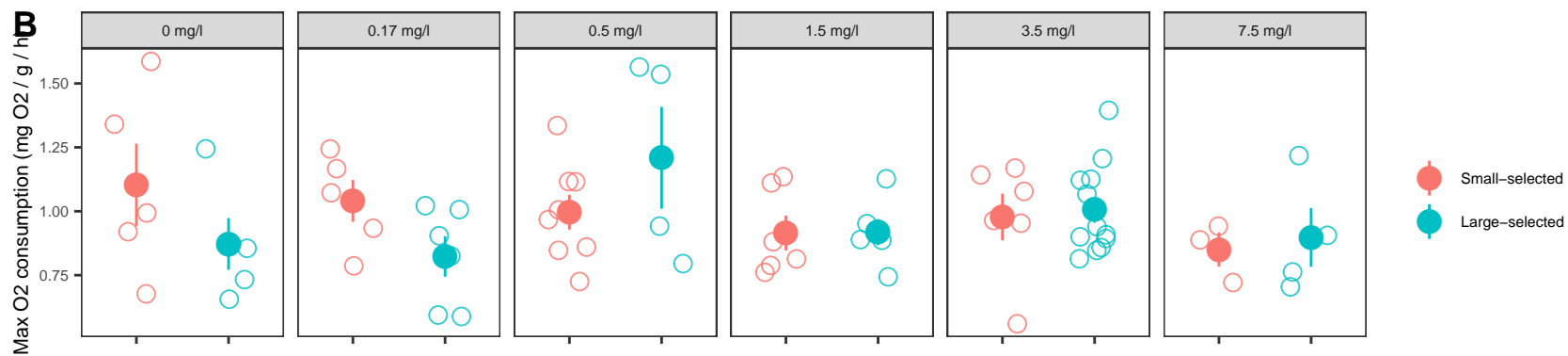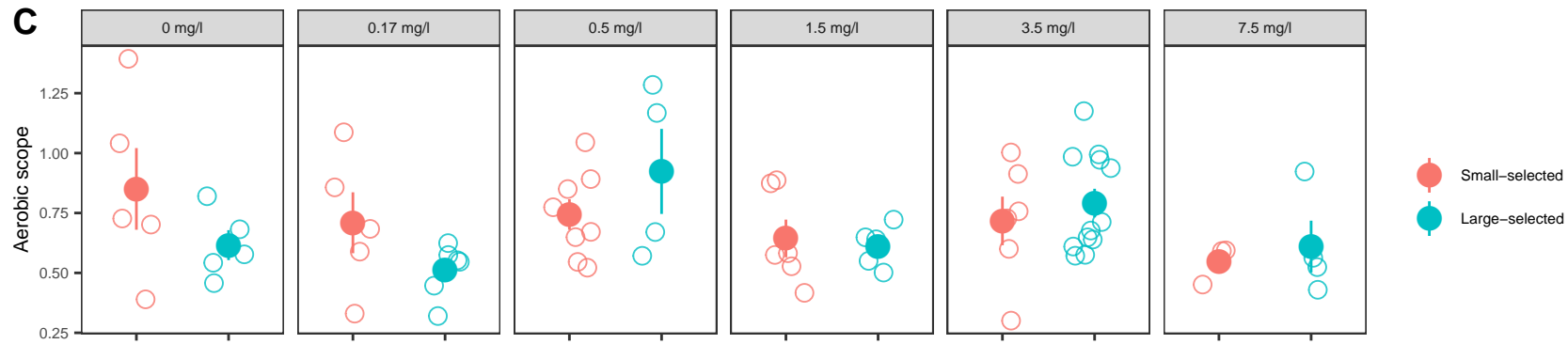

Supplement: Supplementary file 3 — Figure S2 [file ECE3-14-e70134-s001.pdf]
